# Fractional Flow Reserve in the Left Anterior Descending Artery

**DOI:** 10.3390/jcm14155429

**Published:** 2025-08-01

**Authors:** Chang-Ok Seo, Hangyul Kim, Jin-Sin Koh

**Affiliations:** Division of Cardiology, Department of Internal Medicine, Gyeongsang National University School of Medicine and Gyeongsang National University Hospital, Jinju 52727, Republic of Korea; changokseo@gmail.com (C.-O.S.); 13medicine@naver.com (H.K.)

**Keywords:** fractional flow reserve, left anterior descending artery, coronary physiology

## Abstract

Fractional flow reserve (FFR) is a standard physiological index for guiding coronary revascularization, with a threshold of >0.80 typically used to defer intervention. However, due to its distinct anatomical and physiological features, the left anterior descending artery (LAD) often exhibits lower FFR values than non-LAD vessels for lesions of similar angiographic severity. These vessel-specific differences raise concerns about applying a uniform FFR cutoff across all coronary territories. Observational studies indicate that LAD lesions deferred at an FFR of 0.80 may have similar or better outcomes than non-LAD lesions do. LAD lesions also tend to show lower post-percutaneous coronary intervention FFR values, suggesting that vessel specific target thresholds may be more prognostically appropriate. Additionally, some evidence suggests that instantaneous wave-free ratio may offer greater prognostic value than FFR, specifically in LAD lesions, a trend not consistently seen in other arteries. In patients with acute myocardial infarction and multivessel disease, the prognostic relevance of non-culprit lesion FFR may vary by coronary territory, particularly in the LAD. This review outlines the physiological rationale and clinical evidence for vessel-specific interpretation of FFR, with a focus on the LAD, and explores its potential clinical implications and limitations.

## 1. Introduction

Fractional flow reserve (FFR) is a foundational physiological tool in contemporary cardiology, widely used to guide decisions on coronary revascularization. It typically employs a threshold value of 0.80 to determine the functional significance of coronary stenosis [1]. Initially introduced by Pijls and colleagues, FFR assesses lesion-specific ischemia by measuring the ratio of distal coronary pressure to aortic pressure under pharmacologically induced maximal hyperemia [2]. An early validation study established that FFR values above 0.75 reliably correlated with negative ischemia results on noninvasive stress tests, such as exercise electrocardiography, thallium scintigraphy, and dobutamine stress echocardiography [3].

The DEFER trial later adopted 0.75 as the ischemic threshold to prioritize high specificity in guiding percutaneous coronary intervention (PCI) deferral [4]. However, subsequent trials—particularly FAME 1—demonstrated that using a threshold of 0.80 improved sensitivity and better identified functionally significant lesions, especially in intermediate stenoses [5]. FAME 1 also showed improved outcomes with FFR-guided PCI compared to angiography-guided PCI, and FAME 2 confirmed the benefit of FFR-guided PCI over medical therapy in stable coronary artery disease [6]. Consequently, the 0.80 threshold has become the standard for contemporary FFR-guided revascularization.

The left anterior descending artery (LAD) is the most significant coronary vessel, supplying the largest myocardial territory. It is distinctively longer, more tortuous, and more frequently affected by atherosclerotic disease than other coronary arteries [7,8]. These unique anatomical and hemodynamic features expose the LAD to variable shear stress, increased plaque vulnerability, and higher clinical risks. Indeed, clinical studies have consistently shown that the LAD accounts for more than half of all clinically significant coronary events, indicating its critical role in coronary artery disease [9,10].

Traditionally, a uniform FFR cutoff of 0.80 has been applied across all coronary arteries. However, recent evidence shows that the LAD often exhibits lower FFR values than non-LAD vessels do, despite comparable angiographic stenosis severity. This finding raises important questions about the appropriateness of applying a single universal FFR cutoff across different coronary territories.

Given these considerations, growing interest has emerged in the potential benefits of vessel-specific FFR interpretation, particularly for the LAD [11]. This review critically evaluates current evidence on the application and prognostic significance of FFR in LAD versus non-LAD lesions across various clinical scenarios, including lesion deferral, post- PCI assessment, comparisons with instantaneous wave-free ratio (iFR), and the management of non-culprit lesions in patients with acute myocardial infarction (AMI) and multivessel disease (MVD).

## 2. Differences in FFR Measurements in the LAD

The LAD is considered the most clinically significant coronary artery, supplying approximately 50–60% of the left ventricular myocardium. Due to its extensive perfusion territory, even relatively mild lesions in the LAD can have greater clinical implications than similar lesions in other coronary vessels.

**Figure 1:**
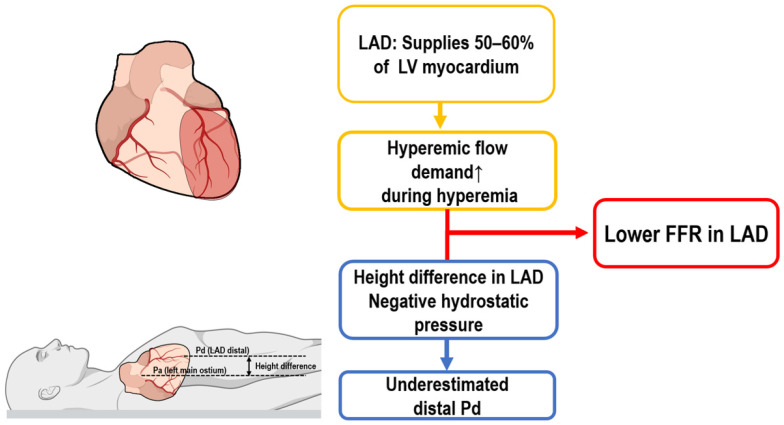
Mechanisms contributing to lower FFR in the LAD. FFR, fractional flow reserve; LAD, left anterior descending artery.

Several unique anatomical and physiological characteristics of the LAD significantly influence FFR measurements. Firstly, due to its large myocardial territory, the LAD has substantially higher hyperemic flow demands than other coronary arteries do, such as the left circumflex (LCx) and right coronary artery (RCA). These increased flow requirements result in greater pressure gradients across stenotic lesions, thereby consistently producing lower FFR values in the LAD compared to those in similarly stenotic lesions in other vessels (Figure 1) [12].

Another critical anatomical factor affecting FFR measurement in the LAD is its spatial orientation, particularly when the patient is in the supine position during testing. In this position, distal segments of the LAD often lie well above the level of the left coronary ostium, creating a vertical hydrostatic pressure difference. This positional disparity leads to an artificial reduction in distal coronary pressure (Pd), thereby lowering measured FFR values irrespective of actual lesion severity (Figure 1). In contrast, the LCx and RCA generally follow more horizontal or downward courses, reducing or even reversing this hydrostatic effect, which may increase Pd and thus elevate FFR values [13,14].

Our computed tomography (CT)-based study demonstrated the clinical significance of accounting for hydrostatic pressure differences (HPDs). Correcting for HPDs increased the mean FFR in LAD lesions from 0.78 to 0.82, reclassifying 22% of lesions from ischemic (FFR ≤ 0.80) to non-ischemic. In comparison, minor decreases were observed in LCx (from 0.87 to 0.85) and RCA (from 0.87 to 0.86) lesions following the same correction, demonstrating the distinct impact of HPD correction on LAD FFR interpretation [15].

Additionally, incomplete hyperemia during intravenous adenosine infusion is another technical factor disproportionately influencing LAD FFR measurements. For example, Matsumoto et al. reported that more than half of LAD lesions initially deemed non-ischemic (FFR > 0.80) by intravenous adenosine infusion were reclassified as ischemic upon reassessment using papaverine, a more potent hyperemic agent [16]. Although this phenomenon is less common in non-LAD vessels, it indicates a source of variability in LAD FFR interpretation.

Similarly, the potential attenuation of adenosine-induced hyperemia by recent caffeine intake remains controversial. While one study suggests that caffeine may blunt the vasodilatory effect of adenosine and alter FFR results in selected patients [17], another found no significant impact when standard intravenous adenosine dosing was used [18]. Although routine caffeine abstention is not currently recommended, a brief intake history may aid interpretation in borderline cases.

Taken together, although incomplete hyperemia induced by intravenous adenosine infusion may occasionally result in artificially elevated FFR values, clinical studies consistently demonstrate that LAD lesions more frequently measure FFR ≤ 0.80 compared to lesions in other coronary arteries, even when angiographic stenosis criteria are not met (Table 1) [19,20,21]. This trend can be attributed to specific features of the LAD, including its extensive myocardial perfusion territory, which imposes higher flow demands, and hydrostatic pressure effects related to its anatomical orientation. Therefore, it remains essential to evaluate how inherently lower LAD FFR values impact clinical decision-making and outcomes in real-world practice.

Despite its widespread use, FFR has several practical limitations. Its reliance on pharmacologic hyperemia may cause patient discomfort and prolong procedure time. Accuracy may be reduced in settings of microvascular dysfunction, unstable hemodynamics, or pressure-wire drift. In such cases, alternative or complementary tools—such as non-hyperemic indices (e.g., iFR), coronary flow reserve (CFR), or intracoronary imaging—may provide additional physiologic insight and support more reliable decision-making.

## 3. FFR-Guided Deferral in LAD vs. Non-LAD Lesions: Insights from Clinical Data

The clinical utility of FFR-guided PCI has been established by pivotal trials such as DEFER and FAME, forming the foundation for current physiology-based revascularization guidelines [5,6,22]. However, an important limitation emerges when these studies are examined through a vessel-specific lens: they primarily report outcomes on a per-patient basis without stratifying results by coronary artery.

For instance, although the DEFER trial included a substantial proportion of LAD lesions (47%), it did not evaluate outcomes based on vessel type. Similarly, FAME 1 and FAME 2 identified functionally significant lesions in multivessel disease (FFR ≤ 0.80) but did not examine whether the prognostic impact of deferral differed by vessel. As a result, whether a uniform FFR threshold carries equal prognostic validity across different coronary arteries—particularly between the LAD and non-LAD—remains an open question.

In the absence of vessel-level data from RCTs, observational registries—despite inherent limitations such as confounding and selection bias—currently offer the most detailed insights into this issue (Table 2). While FFR is a widely accepted physiological index, its application across studies is not uniform. To account for technical heterogeneity, we provide a summary of FFR methodology from key studies in Supplementary Table S1.

In the J-CONFIRM Registry, a prospective multicenter study conducted in Japan to evaluate FFR-based deferral of revascularization in patients with stable coronary artery disease (CAD), vessel-specific analysis of angiographically intermediate lesions deferred based on FFR revealed important differences in clinical outcomes [23]. Deferral of LAD lesions was associated with a significantly lower risk of target vessel failure (TVF) (hazard ratio [HR], 0.42; *p* = 0.003), whereas deferral of RCA lesions was associated with an increased risk of TVF (HR, 1.78; *p* = 0.042). Deferral of LCx lesions showed no significant impact on outcomes (Table 2). Notably, the FFR values at the time of deferral were not bound to a strict cutoff of 0.80 but instead reflected operator discretion in real-world clinical practice. Despite this variability, these findings suggest that physiology-guided deferral of LAD lesions may be associated with better outcomes than deferral in other coronary territories.

A recent vessel-specific analysis from Australia retrospectively evaluated outcomes in 867 lesions (733 patients) deferred based on negative FFR (>0.80) over a 3-year follow-up [24]. Mean FFR values were significantly lower in LAD lesions (0.82) than in LCx (0.88) and RCA (0.88). Despite this difference, 3-year target lesion failure rates were similar across vessels (LAD 10.2%, LCx 11.5%, RCA 11.0%), with no significant differences after multivariate adjustment. Moreover, the incidence of target lesion myocardial infarction was numerically lower in LAD lesions (1.45%) than in LCx (4.09%) and RCA (2.05%), although this did not reach statistical significance.

The IRIS-FFR registry, a large-scale observational study evaluating outcomes after FFR-based deferral of PCI, analyzed over 8600 lesions during a mean follow-up of 1.9 years [25]. The study demonstrated a clear inverse relationship between FFR values and major adverse cardiovascular events (MACEs). Lesions with FFR ≤ 0.75 benefited significantly from revascularization, whereas those with FFR ≥ 0.76 showed comparable outcomes when deferred. Importantly, vessel-specific analysis revealed similar MACE incidence rates per lesion-year across coronary arteries: LAD (1.7%), RCA (1.47%), and LCx (1.25%), suggesting comparable prognostic safety of FFR-based deferral across all three vessels.

The Korean FFR Registry is a multicenter observational study evaluating 3-year clinical outcomes of deferred coronary lesions according to functional severity assessed by FFR [26]. Among 781 deferred lesions, LAD lesions were more frequently represented in the lower FFR strata. Over a three years follow-up, LAD deferral was associated with a lower risk of MACEs compared to non-LAD deferral; however, this difference did not reach statistical significance after multivariate adjustment (HR 0.56; confidence interval [CI], 0.31–1.01; *p* = 0.052). A similar trend was observed in the subgroup of lesions with FFR > 0.80, where LAD deferral showed favorable outcomes in univariate analysis (HR 0.45; *p* = 0.012), but this association also lost statistical significance after multivariate adjustment (HR 0.57; *p* = 0.095). These findings suggest a potential clinical benefit from FFR-based deferral of LAD lesions, although definitive evidence remains limited.

The Mayo FFR Registry, a large single-center study, demonstrated a significant association between FFR-guided deferral of PCI and improved long-term outcomes [27]. In this cohort, LAD lesions represented the majority of evaluated cases (65.4%), with subsequent PCI performed in only 5.4% of deferred LAD lesions. Similar rates of revascularization were observed for deferred lesions in the LCx (6.2%) and RCA (6.9%), reinforcing the consistent safety and efficacy of FFR-guided deferral irrespective of coronary vessel location.

Taken together, clinical evidence consistently indicates that although LAD lesions typically yield lower FFR values, their outcomes following FFR-based deferral are comparable or even more favorable than those of non-LAD lesions. Given that the LAD is traditionally considered a high-risk vessel due to its extensive myocardial territory and greater plaque burden, the observation of similar or better outcomes with FFR-based deferral is particularly noteworthy. This apparent paradox may be attributed to intrinsic LAD features that systematically lower FFR values, potentially overestimating ischemic severity. Consequently, among lesions deferred at similar FFR thresholds, LAD lesions may represent less anatomically or functionally severe disease than non-LAD lesions. In this context, the inherent risk of LAD lesions may be offset by the physiologic tendency to overestimate their severity, resulting in comparable clinical outcomes. While these registries and post hoc analyses provide valuable real-world insights, they are inherently limited by confounding, selection bias, and the absence of vessel-level randomization. As such, these findings should be interpreted with caution, and further prospective randomized studies are warranted to confirm the prognostic value of vessel-specific FFR thresholds in routine clinical practice.

**Figure 2:**
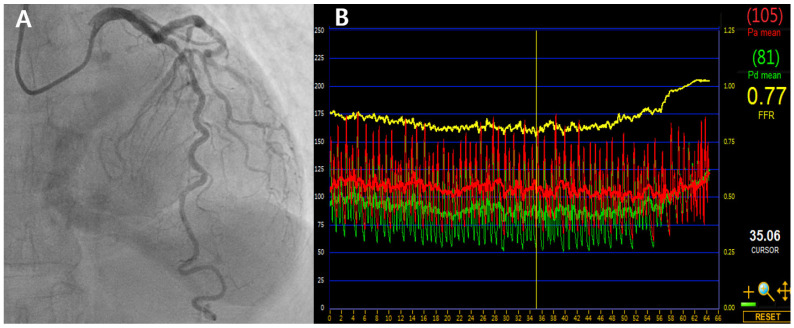
Coronary angiography and FFR tracing of a borderline LAD lesion. (**A**) Angiogram showing 70% stenosis in the proximal to mid segment of the LAD artery. (**B**) FFR measured at 0.77 was adjusted to 0.81 using a validated LAD-specific hydrostatic correction model, supporting deferral of PCI. LAD, left anterior descending artery; FFR, fractional flow reserve; PCI, percutaneous coronary intervention.

These observations raise the question of how borderline FFR values should be interpreted in real-world practice. The following case in Figure 2 illustrates how LAD-specific anatomical and physiological features can influence treatment decisions when the FFR is near the conventional threshold.

A 68-year-old man with stable angina and diabetes presented with exertional chest discomfort. Coronary angiography revealed a 70% intermediate stenosis in the proximal to mid segment of the LAD artery (Figure 2A). FFR measured using intravenous adenosine was 0.77 (Figure 2B)—technically ischemic by conventional criteria. However, based on our previously validated LAD-specific height correction model, which accounts for an average hydrostatic offset of +0.04, the corrected FFR was estimated to be approximately 0.81. No regional wall motion abnormalities were noted on stress echocardiography. Given these anatomical and physiologic LAD-specific considerations, PCI was deferred and the patient was managed with optimal medical therapy. He remained asymptomatic and free of major adverse cardiac events at 6-year follow-up.

This case exemplifies how applying vessel-specific insights, such as hydrostatic correction, may reclassify borderline LAD lesions and help avoid unnecessary intervention. Conversely, non-LAD lesions with marginally higher FFR may still carry more risk if deferred based solely on a rigid threshold—further supporting the need for individualized physiological assessment. This phenomenon reflects a “physiological paradox”, where the LAD’s structural characteristics—such as its larger subtended territory and vertical course—can lower FFR values without corresponding to true ischemic burden. This may help explain why LAD lesions, despite lower FFRs, often show outcomes comparable to or better than non-LAD lesions, further supporting the need for individualized physiological assessment.

## 4. Post-PCI FFR: LAD vs. Non-LAD

Post-PCI FFR has emerged as an essential tool for evaluating the physiological success of coronary revascularization. Traditionally, angiographic results have guided procedural success; however, growing evidence indicates that significant residual ischemia may persist even after angiographically successful procedures. For instance, the DEFINE-PCI study reported residual ischemia (defined as iFR < 0.89) in 24% of patients despite seemingly successful angiographic outcomes [28]. These findings highlight the limitations of relying solely on angiographic assessments and support incorporating post-PCI FFR evaluations.

Studies suggest varied optimal post-PCI FFR thresholds, generally ranging from 0.80 to 0.96 [29,30,31]. A recent meta-analysis found a threshold of ≤0.80 associated with increased risks of cardiac death and target vessel myocardial infarction, while a cutoff of ≤0.86 correlated with higher rates of TVF [32].

A post hoc analysis of the DKCRUSH VII trial, one of the earliest studies to examine vessel-specific differences in prognostic post-PCI FFR values, showed that lower post-PCI FFR values were independently associated with a higher risk of TVF, with values ≤ 0.88 linked to approximately twice the risk over a 3-year follow-up [33]. Notably, a higher FFR cutoff of 0.905 offered superior prognostic accuracy, specifically for LAD lesions, suggesting that more stringent physiological targets may be required for optimal outcomes in this territory. However, as the DKCRUSH VII trial primarily involved high-risk bifurcation lesions, the applicability of these thresholds to non-bifurcation or routine lesions remains limited.

Hwang et al. conducted a prospective multicenter registry involving 835 patients from nine centers across Korea and Japan, evaluating post-PCI FFR [34]. They found significantly lower median post-PCI FFR values in LAD and non-LAD lesions (0.85 vs. 0.92; *p* < 0.001), despite similar angiographic outcomes. Vessel-specific FFR thresholds for predicting 2-year TVF were identified as 0.82 for LAD and and 0.88 for non-LAD lesions, respectively. Patients below these thresholds experienced significantly higher TVF rates (10.9% vs. 2.5%, HR 4.8, *p* = 0.001 for LAD lesions; and 8.0% vs. 1.9%, HR 6.0, *p* < 0.01 for non-LAD lesions), while angiographic stenosis severity did not predict outcomes. Receiver operating characteristic (ROC) analysis yielded AUC values of 0.70 for LAD and 0.76 for non-LAD lesions. While these values reflect moderate statistical discrimination, their clinical utility remains limited and insufficient to support definitive vessel-specific thresholds without further validation.

Collet et al. conducted an individual patient-level meta-analysis to assess the prognostic significance of post-PCI FFR stratified by coronary artery. Analyzing 3336 vessels from 2760 patients across nine studies, they found that post-PCI FFR values were significantly lower in the LAD (mean 0.86) compared to the LCx and RCA (both mean 0.93 and 0.91, respectively). Importantly, while lower FFR values were associated with higher rates of TVF, the predictive accuracy of post-PCI FFR differed by vessel. The AUC for TVF prediction was poor for LAD (AUC 0.52) and moderate for non-LAD vessels (AUC 0.66). These findings suggest the limitations of applying a uniform FFR cutoff across all coronary territories and support the rationale for vessel-specific physiological assessment after PCI [35] (Table 3).

Successful revascularization is generally expected to result in post-PCI FFR values well above conventional thresholds. However, procedural factors such as distal microembolism and transient hyperemia can reduce hyperemic flow, producing blunted FFR responses and artificially elevated post-PCI FFR values. Despite these procedural limitations, multiple studies—excluding those specifically addressing bifurcation lesions—consistently show systematically lower post-PCI FFR values in LAD lesions compared to non-LAD lesions. This finding is consistent with the tendency for LAD lesions to exhibit inherently lower FFR both before and after PCI. Additionally, vessel-specific post-PCI FFR thresholds further support the rationale for adopting tailored FFR targets for LAD lesions. However, clear clinical benefit from such tailored thresholds remains unproven, as reflected by the limited prognostic accuracy (AUC 0.52 in meta-analysis) of LAD-specific post-PCI FFR values [35]. Therefore, additional studies are necessary to establish the clinical utility and practical applicability of vessel-specific post-PCI FFR thresholds in routine clinical practice.

## 5. iFR vs. FFR in LAD Deferral

The iFR is a non-hyperemic physiologic index that measures the pressure gradient across a coronary stenosis during a specific wave-free period in diastole, eliminating the need for pharmacologic vasodilation. This feature makes iFR a simpler and faster alternative to FFR in routine practice. Two large-scale RCTs—DEFINE-FLAIR and iFR-SWEDEHEART—demonstrated the non-inferiority of iFR-guided revascularization compared to FFR-guided strategies in terms of MACE, leading to a Class I recommendation in contemporary guidelines [36,37]. Notably, iFR was also associated with shorter procedural time and fewer symptoms during assessment. However, discordance between iFR and FFR has been observed in approximately 15–20% of angiographically similar lesions, more frequently in those located in the LAD [38], raising questions about which index more accurately reflects lesion-specific ischemic risk in certain cases.

Recently, a sub-analysis of the DEFINE-FLAIR trial reported an intriguing finding suggesting that iFR may provide superior prognostic value over FFR, specifically in LAD lesions [39]. Among patients with LAD disease, those whose lesions were deferred based on iFR ≥ 0.89 (*n* = 451) or FFR ≥ 0.80 (*n* = 421) were followed for 1 year. The iFR-guided group showed a significantly lower rate of MACEs compared to the FFR-guided group (2.44% vs. 5.46%; HR 0.46; *p* = 0.04). No significant difference was observed in non-LAD lesions, suggesting a potential advantage of iFR-guided deferral in the LAD. Several studies suggest that iFR may better reflect true coronary physiology than FFR, owing to its stronger correlation with CFR—a well-established predictor of long-term clinical outcomes. CFR is defined as the ratio of maximal hyperemic to resting coronary blood flow, providing an integrated assessment of both epicardial and microvascular function. The JUSTIFY-CFR [40] and IDEAL studies [41] showed that iFR is more closely aligned with CFR than FFR, particularly in intermediate lesions where decision-making is most challenging. This physiologic concordance may explain the superior prognostic discrimination of iFR in this subset.

Other non-hyperemic indices, such as Resting Full-cycle Ratio (RFR) and Diastolic Pressure Ratio (dPR), have also been introduced. However, vessel-specific validation—particularly in the LAD—is still limited, and robust outcome data are lacking. Therefore, this review focuses on iFR and FFR, which are currently best supported by clinical evidence in this context.

Nevertheless, emerging long-term data advise caution. The 5-year outcomes from the DEFINE-FLAIR trial raise concerns regarding the long-term safety of iFR-guided strategies [42]. While overall MACE rates were comparable to FFR, patients who underwent revascularization based on iFR experienced a significantly higher MACE rate (24.6% vs. 19.2%; HR 1.36; *p* = 0.01) and a trend toward increased all-cause mortality (HR 1.18; *p* = 0.06). In contrast, outcomes in deferred patients were similar between groups, suggesting potential limitations in iFR’s ability to identify prognostically significant lesions when intervention is pursued. This signal was reinforced by a meta-analysis incorporating the SWEDEHEART registry, which found a significantly higher all-cause mortality with iFR (HR 1.38; 95% CI, 1.12–1.69; *p* < 0.01), largely driven by non-cardiovascular deaths [43]. Although the mechanism remains unclear, reduced revascularization in ACS patients may be contributory. In light of the emerging concerns from long-term outcome data, even deferral decisions based on iFR in LAD may warrant a more cautious approach until further robust, long-term evidence becomes available.

## 6. Non-Culprit Lesion FFR in AMI with MVD: LAD vs. Non-LAD

In patients with AMI and MVD, the optimal management of non-culprit lesion remains clinically challenging. Five major RCTs—DANAMI-3-PRIMULTI [44], COMPARE-ACUTE [45], FULL REVASC [46], FRAME-AMI [47], and FLOWER-MI [48]—have evaluated the efficacy of FFR-guided complete revascularization (CR) in this context. Among these, DANAMI-3-PRIMULTI, COMPARE-ACUTE, and FRAME-AMI demonstrated significant reductions in MACEs with FFR-guided CR than with culprit-only PCI, whereas FULL REVASC and FLOWER-MI did not demonstrate clear superiority. These inconsistent findings prompt reconsideration of the appropriateness of uniformly applying an FFR threshold of 0.80 in this clinical setting and further suggest that vessel-specific thresholds may be required to optimize prognostic accuracy and clinical decision-making.

A vessel-level subanalysis of the COMPARE-ACUTE trial provided key insights into how FFR values relate to clinical outcomes in patients with AMI and MVD [49]. Lesions associated with adverse events had significantly lower FFR values across all major coronary arteries. Notably, LAD lesions associated with clinical events exhibited the lowest median FFR (0.77 vs. 0.82; *p* = 0.002) compared to the ones in LCx (0.83 vs. 0.89; *p* < 0.001) and RCA (0.82 vs. 0.87; *p* = 0.012) underscoring the distinct physiological and prognostic implications of LAD involvement (Table 4). 

The FRAME-AMI substudy evaluated the clinical impact of FFR-guided PCI in patients with AMI and MVD, with a specific focus on the anatomical location of non-culprit lesions [50]. Among the 562 patients enrolled, 309 (55.0%) had non-culprit lesions in the LAD, and 253 (45.0%) had lesions in non-LAD territories. In the LAD group, FFR-guided PCI significantly reduced the incidence of the primary composite outcome—death, myocardial infarction, or repeat revascularization—compared to angiography-guided PCI (5.7% vs. 14.3%; *p* = 0.010). In contrast, for non-LAD lesions, FFR guidance showed a trend toward benefit, but the difference did not reach statistical significance (7.4% vs. 14.5%; *p* = 0.081). Anatomically, LAD lesions were more severe, with longer lesion lengths, greater diameter stenosis (mean 71.5%), and lower average FFR values (0.77 vs. 0.83). Notably, 62.1% of LAD lesions had FFR ≤ 0.80, compared to 36.8% of non-LAD lesions.

The FULL REVASC trial compared FFR-guided complete revascularization with culprit-only PCI in patients presenting with STEMI or high-risk NSTEMI [46]. Over a median follow-up of 4.8 years, the study found no significant difference in clinical outcomes between the two strategies. When analyzed by individual coronary arteries, FFR-guided revascularization did not demonstrate a clear benefit: HRs were 0.91, 0.85, and 1.16 for the LAD, LCx, and RCA, respectively, with no significant differences observed in any vessel subgroup. Even in proximal LAD lesions—typically regarded as high-risk—the event rate was numerically lower with FFR guidance (HR 0.81), but this, too, lacked statistical significance.

Taken together, the data raise the important consideration that the conventional FFR threshold of ≤0.80 may not equally reflect ischemic risk across all coronary territories. In particular, LAD lesions—due to their anatomical dominance and higher prevalence of physiologically significant disease—may warrant a more discriminating or vessel-specific physiological threshold. These findings support the rationale for tailored ischemia-guided strategies that account for vessel territory in clinical decision-making for the patients with AMI and MVD.

Nonetheless, interpreting FFR in non-IRA lesions during the acute phase of AMI remains challenging, primarily due to dynamic and heterogeneous physiological responses. It remains unclear whether microvascular dysfunction—commonly observed in AMI—is confined to the culprit artery or involves the entire myocardial circulation [51,52,53]. Instability in both resting and hyperemic coronary flow during the acute setting further complicates the reliability of FFR measurements. Moreover, these acute physiological changes may vary considerably across coronary territories, with some vessels potentially more affected than others—a variability that remains incompletely characterized. Consequently, applying a uniform FFR cutoff without accounting for vessel-specific physiological contexts may result in suboptimal clinical decisions. In STEMI patients, especially those with hemodynamically unstable conditions or anatomically complex non-culprit lesions, immediate FFR measurement in index procedure may be suboptimal due to physiologic variability and impaired microvascular function. In such cases, delayed assessment after clinical stabilization—typically days or weeks later—may yield more reliable physiologic data and support safer decision-making regarding staged PCI. Moreover, under unstable conditions, vessel-specific physiologic variability may be amplified, further complicating the use of fixed or even vessel-adjusted FFR thresholds in the acute setting. Accordingly, future research should focus on delineating vessel-specific physiological characteristics during AMI to inform distinct FFR thresholds that could enhance both decision-making and therapeutic precision.

## 7. Is Vessel-Specific FFR Targeting Feasible and Justified?

Accumulating evidence suggesting vessel-specific variability in FFR values—particularly within the LAD—has led to increasing interest in moving beyond a single universal threshold toward territory-specific cutoffs. The rationale is compelling: structural and physiological differences between coronary arteries may systematically influence FFR measurements and their prognostic meaning.

However, several critical questions remain. First, while post hoc and observational studies suggest that vessel-specific thresholds may enhance prognostic discrimination, these findings are not yet supported by prospective RCTs. The absence of level I evidence makes it difficult to justify a shift in clinical practice, particularly when current guideline recommendations remain anchored to a uniform threshold of 0.80. Moreover, although concerns have been raised regarding the adequacy of applying this universal cutoff—especially in the LAD—accumulated observational data do not consistently indicate worse outcomes when deferral is based on FFR >0.80 in this vessel. Indeed, the comparable distribution of adverse events across coronary territories in several studies calls into question not only the clinical utility of vessel-specific thresholds, but also the necessity of conducting dedicated RCTs to validate them. This reflects a broader tension between the pursuit of physiological precision and the pragmatic simplicity of standardized practice, suggesting the need for caution before revising established paradigms in the absence of definitive outcome-based evidence.

**Figure 3:**
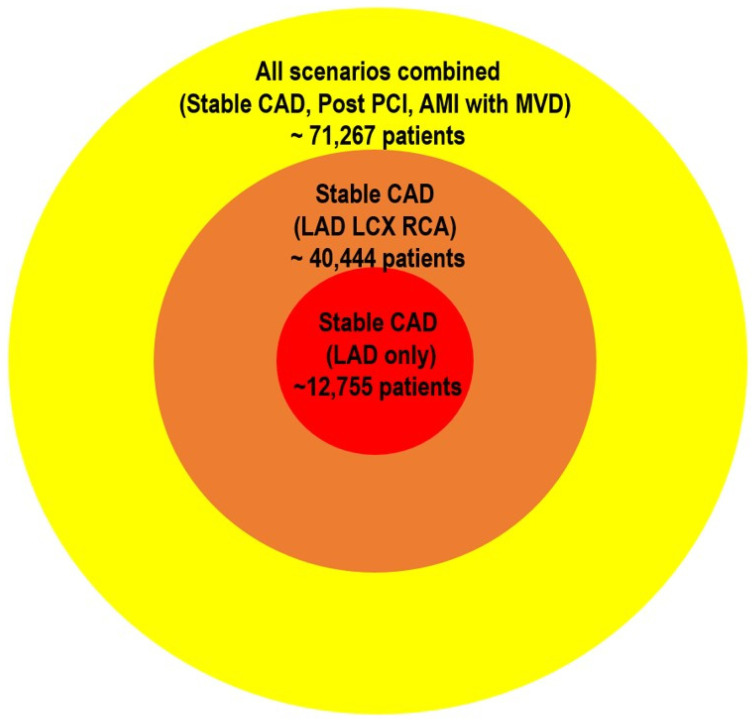
Required sample sizes for vessel-specific FFR RCTs. The estimated sample sizes illustrate the substantial logistical demands of conducting RCTs to validate vessel-specific FFR thresholds. Sample size calculations were based on conservative—highest observed—event rates from previously published vessel-level studies: stable CAD from J-CONFIRM [20]; AMI with MVD from COMPARE-ACUTE [45] and FULL REVASC [46]; and post-PCI FFR scenarios from Hwang et al. [34] and Collet et al. [35]. A standard two-group proportion comparison approach was used, assuming a statistical power of 80%, significance level of 5%, and a relative risk reduction of 20%. Altogether, these scenarios underscore the immense patient numbers required, highlighting the practical infeasibility of dedicated RCTs for establishing vessel-specific FFR thresholds. FFR, fractional flow reserve; RCT, randomized clinical trial; LAD, left anterior descending artery; CAD, coronary artery disease; LCx, left circumflex; RCA, right coronary artery; MVD, multivessel disease; AMI, acute myocardial infarction; PCI, percutaneous coronary intervention.

Second, validating vessel-specific FFR cutoffs would require prohibitively large, dedicated outcome trials. Even when using the most conservative—that is, the highest observed—event rates from prior vessel-level studies, the estimated sample sizes remain immense. For example, in stable CAD, approximately 12,755 patients would be required for the LAD [20], 11,104 for the RCA, and 16,585 for the LCx, totaling over 40,000 patients. Post-PCI studies would add another 19,000 patients [32,33], and AMI with MVD would require roughly 12,000 more [45,46]. Altogether, these scenarios would necessitate over 70,000 participants, underscoring the practical infeasibility of conducting separate RCTs to define FFR thresholds for each coronary vessel (Figure 3).

Third, even if vessel-specific FFR thresholds offer superior prognostic value, their clinical applicability remains uncertain. One particular area illustrating this issue is the interpretation of post-PCI FFR values. For example, proposed post-PCI cutoffs vary widely—from 0.80 to 0.96 [27,28,29]—raising concerns that no single value can reliably represent the full spectrum of individual lesion risk. This variability reflects the limitations of relying on fixed thresholds in a physiologically continuous domain. Moreover, a recent meta-analysis of nine pivotal studies, encompassing 3336 individual post-PCI FFR measurements, showed limited prognostic accuracy for predicting adverse outcomes in LAD lesions, with a notably low AUC of 0.52 [33]. This variability highlights the difficulty in establishing a universally applicable numerical threshold, suggesting that refining cutoffs or merely increasing data volume may not meaningfully improve patient outcomes. Therefore, there is a need to fundamentally reassess how physiological measurements are interpreted clinically, moving beyond reliance on numerical thresholds alone in each vessel.

Lastly, the advancement of coronary artery CT derived FFR, along with other image-based physiologic assessment methods such as quantitative flow ratio (QFR), is reshaping the landscape of coronary physiology [54,55,56,57,58]. These modalities—many of which are increasingly integrated with artificial intelligence (AI) and advanced computational modeling—enable noninvasive, rapid, and lesion-specific functional evaluation without requiring pressure wires or pharmacologic hyperemia. Although originally developed using invasive pressure-based FFR data, these technologies now aim to overcome the limitations of traditional FFR by offering a more precise, patient-specific depiction of coronary physiology. Future research may demonstrate that such AI-enhanced, image-based methods provide a more scalable alternative to vessel-specific FFR thresholds, with the potential to enhance both diagnostic accuracy and clinical outcomes.

## 8. Conclusions

FFR remains one of the most extensively validated physiologic tools for assessing the functional significance of coronary stenosis, with demonstrated utility across diverse clinical settings. However, as our understanding of coronary physiology continues to evolve, it is increasingly clear that a single numeric cutoff may not adequately capture the complexity of vessel-specific anatomy, lesion characteristics, or clinical context.

Given the substantial practical and logistical barriers to defining precise vessel-specific thresholds through RCTs, future clinical decision-making should leverage existing clinical data, advanced imaging technologies, and AI-driven analyses to deepen our understanding and application of vessel-specific FFR. Such an integrative strategy may enable more personalized interpretation of physiological data and ultimately improve both diagnostic accuracy and therapeutic outcomes.

Moving forward, clinical practice should emphasize individualized integration of anatomical and physiological assessments rather than relying on fixed numeric thresholds. The future of coronary physiology lies not in defining another cutoff, but in refining how we interpret and apply these measurements. Precision does not reside in the number itself, but in the meaning we assign to it.

## Figures and Tables

**Table 1:** Prevalence of physiologically significant lesions by vessel in clinical studies.

STUDY	Angiographic Stenosis Criteria (QCA)	LAD (FFR ≤ 0.80, %)	LCx (FFR ≤ 0.80, %)	RCA (FFR ≤ 0.80, %)
RIPCORD [19]	<50%	18.0%	13.5%	8.5%
FLAVOUR [20]	<60%	82.2%	3.4%	14.4%
CVIT-DEFER [21]	<75%	33.4%	8.7%	9.7%

QCA: quantitative coronary angiography; LAD: left anterior descending artery; LCx: left circumflex artery; RCA: right coronary artery; FFR: fractional flow reserve.

**Table 2:** Key Clinical studies on FFR-guided deferral with LAD-specific findings.

Study	Study Type	LAD-Specific Outcomes	Implications for LAD
DEFER [22]	Pioneering RCT	LAD lesions (>50%), no specific outcomes reported	Indirect safety evidence
FAME 1/2 [5,6]	Large-scale RCTs	No vessel-specific outcomes	No direct LAD-specific data
J-CONFIRM [23]	Multicenter Registry	LAD deferral: ↓TVF (HR 0.42, *p* = 0.003) RCA deferral: ↑TVF (HR 1.78, *p* = 0.042) LCx deferral: NS	Supports safety of LAD deferral
Ekmejian et al. [24]	Multicenter Registry	Mean FFR: LAD lowest (0.82), LCx (0.88), RCA (0.88) TLF similar (10.2% LAD vs. 11.5% LCx, 11.0% RCA, NS) TLMI lower in LAD (1.45%, NS)	Lower FFR but comparable outcomes in LAD
IRIS-FFR [25]	Multicenter Registry	Annual MACEs: LAD 1.7%, LCx 1.47%, RCA 1.25%	Similar outcomes across vessels
Korean FFR [26]	Multicenter Registry	LAD deferred: lower MACE trend vs. non-LAD (HR 0.57, NS).	Possible LAD benefit (limited evidence)
Mayo FFR [27]	Single-center Registry	Similar PCI rates after deferral (LAD 5.4%, LCx 6.2%, RCA 6.9%)	Similar outcomes across vessels

FFR = fractional flow reserve; RCT = randomized clinical trial; NS = not significant; TVF = target vessel failure; TLF = target lesion failure; TLMI = target lesion myocardial infarction; LAD = left anterior descending artery; LCx = left circumflex artery; RCA = right coronary artery; MACEs = major adverse cardiovascular events; PCI = percutaneous coronary intervention; HR = hazard ratio.

**Table 3:** Post-PCI FFR in clinical studies.

Study	LAD Post-PCI Cutoff	Non-LAD Post-PCI Cutoff	Key Findings	Limitations
DKCRUSH VII [33]	0.905	0.88	Higher cutoff for LAD improved prediction; Lower success rate in LAD (59% vs. 85.7%)	High-risk bifurcation lesions
Hwang et al. [34]	0.82	0.88	Lower post-PCI FFR in LAD (0.85 vs. 0.92); TVF in LAD (10.9% vs. 2.5%, HR 4.8), non-LAD (8.0% vs. 1.9%, HR 6.0); AUC: LAD 0.70, non-LAD 0.76	Observational registry
Collet et al. [35]	0.86	0.93 (LCX 0.93 + RCA 0.91)	Lower FFR and prognostic accuracy in LAD; (AUC: 0.52 LAD vs. 0.66 Non-LAD)	Post hoc meta-analysis

PCI = percutaneous coronary intervention; FFR = fractional flow reserve; AUC = area under the curve; LAD = left anterior descending artery; LCX = left circumflex artery; RCA = right coronary artery.

**Table 4:** Non-culprit lesion outcomes of FFR-guided PCI in AMI with MVD.

Trial	Main Result	Vessel-Level Findings (LAD)	Key Point
COMPARE-ACUTE [49]	FFR↓ in event lesions (vs. no event)	FFR in event lesions vs. no event LAD: 0.77 vs. 0.82 (*p* = 0.002) LCx: 0.83 vs. 0.89 (*p* < 0.001) RCA: 0.82 vs. 0.87 (*p* = 0.013)	Lower FFR linked to events; LAD lowest
FRAME-AMI [50]	MACEs: Total: 6.3% (FFR) vs. 14.4% (Angio), *p* = 0.001 LAD: 5.7% (FFR) vs. 14.3% (Angio), *p* = 0.001 Non-LAD: 7.4% (FFR) vs. 14.5% (Angio), *p* = 0.081	LAD vs. Non-LAD Mean FFR: 0.77 vs. 0.83 FFR ≤ 0.80: 62.1% vs. 36.8%	Clear FFR benefit in LAD
FULL REVASC [46]	No difference in MACEs over median 4.8 years	HR for MACEs: LAD 0.91, LCx 0.85, RCA 1.16; proximal LAD HR 0.81 (NS)	No FFR benefit overall or by vessel

AMI = acute myocardial infarction; MVD = multivessel disease; HR = hazard ratio; LAD = left anterior descending artery; LCx = left circumflex artery; RCA = right coronary artery; NS = not significant; MACEs = major adverse cardiovascular events; PCI = percutaneous coronary intervention; FFR = fractional flow reserve.

## Supplementary Materials

The following supporting information can be downloaded at: https://www.mdpi.com/article/10.3390/jcm14155429/s1, Supplementary Table S1. FFR Methodology in Key Studies.

## Abbreviations

AI	Artificial intelligence
AMI	Acute myocardial infarction
CAD	Coronary artery disease
CFR	Coronary flow reserve
CR	Complete revascularization
FFR	Fractional flow reserve
HPDs	Hydrostatic pressure differences
iFR	Instantaneous wave-free ratio
LAD	Left anterior descending artery
LCx	Left circumflex
MVD	Multivessel disease
PCI	Post-percutaneous coronary intervention
Pd	Distal coronary pressure
QFR	Quantitative flow ratio
RCA	Right coronary artery
