# Supplementary material for: Fractional Flow Reserve in the Left Anterior Descending Artery
Source: J Clin Med. 2025 Aug 1;14(15):5429. doi: 10.3390/jcm14155429 (PMC12347470; doi:10.3390/jcm14155429)
Supplement: Supplementary file 1 [file jcm-14-05429-s001.zip › jcm-3718416-supplementary.pdf]

Supplementary Table S1. FFR methodology in key studies

Study Name	Wire Used	Sensor Location	Nitrate Used	Adenosine Route	AV Block Protocol
DEFER	Pressure wire (Radi Medical System)	Distal to stenosis	Not addressed	IV infusion	Not addressed
FAME	Pressure wire (Radi Medical System)	Not addressed	Not addressed	IV infusion	Not addressed
Korean FFR	PressureWire™ (Abbott) or Volcano™ pressure wire	Not addressed	Not addressed	IV + IC	Not addressed
Mayo FFR	PressureWire™ (Abbott) or Volcano™ pressure wire	Distal to stenosis	Not addressed	IV + IC	Not addressed
J-CONFIRM	PressureWire™ (Abbott)	Distal to stenosis	Yes	IV + IC	Not addressed
IRIS-FFR	Commercially available pressure wire	Distal to target lesion	Yes	IV infusion	Not addressed
DEFINE-PCI	Prestige Guide Wire PLUS or Verrata guide wire, Philips/Volcano	Mid-to-distal vessel	Yes	IV + IC	Not addressed
Hwang et al.	PressureWire™ (Abbott) or Volcano™ pressure wire	Distal segment	Yes	IV + IC	Cases with complete AV block excluded
Collet et al.	Mixed	Standardized	Yes	IV + IC	Not addressed
COMPARE-ACUTE	PressureWire™ (St.Jude medical)	Not addressed	Yes	IV + IC	Not addressed
FRAME-AMI	PressureWire™ (Abbott) or Volcano™ pressure wire	Not addressed	Yes	IV + IC	Not addressed
FULL-REVASC	Not addressed	Not addressed	Not addressed	Not addressed	Not addressed

AV: atrioventricular, IV: intravenous, IC intracoronary.
